# Transition from exponential to linear photoautotrophic growth changes the physiology of *Synechocystis* sp. PCC 6803

**DOI:** 10.1007/s11120-016-0329-8

**Published:** 2017-01-20

**Authors:** R. M. Schuurmans, J. C. P. Matthijs, K. J. Hellingwerf

**Affiliations:** 1 Molecular Microbial Physiology Group, Swammerdam Institute for Life Sciences, University of Amsterdam, Amsterdam, The Netherlands; 2 Aquatic Microbiology, Institute for Biodiversity and Ecosystem Dynamics, University of Amsterdam, Amsterdam, The Netherlands

**Keywords:** Batch culture, Cyanobacteria, Molecular physiology, PSI/PSII ratio, State transition

## Abstract

Phototrophic microorganisms like cyanobacteria show growth curves in batch culture that differ from the corresponding growth curves of chemotrophic bacteria. Instead of the usual three phases, i.e., lag-, log-, and stationary phase, phototrophs display four distinct phases. The extra growth phase is a phase of linear, light-limited growth that follows the exponential phase and is often ignored as being different. Results of this study demonstrate marked growth phase-dependent alterations in the photophysiology of the cyanobacterium *Synechocystis* sp. PCC 6803 between cells harvested from the exponential and the linear growth phase. Notable differences are a gradual shift in the energy transfer of the light-harvesting phycobilisomes to the photosystems and a distinct change in the redox state of the plastoquinone pool. These differences will likely affect the result of physiological studies and the efficiency of product formation of *Synechocystis* in biotechnological applications. Our study also demonstrates that the length of the period of exponential growth can be extended by a gradually stronger incident light intensity that matches the increase of the cell density of the culture.

**Electronic supplementary material:**

The online version of this article (doi:10.1007/s11120-016-0329-8) contains supplementary material, which is available to authorized users.

## Introduction

Successful genetic engineering of cyanobacteria for the synthesis of defined (commodity) products in biotechnological applications has been amply demonstrated during the past few years (Angermayr et al. 2012; Dexter and Fu 2009; Nobles and Brown 2008; Savakis et al. 2013). These achievements call for mass cultivation of cyanobacteria; they also initiated this study on the potential growth phase dependency of the photo-physiological properties of the cyanobacterium *Synechocystis* sp. PCC 6803 (hereafter *Synechocystis*). Different from the general growth pattern of chemoheterotrophic bacteria in batch culture, which is characterized by three successive phases: lag phase, exponential growth phase, and stationary phase, respectively, batch growth of phototrophs shows an additional distinctive phase change, i.e., from exponential growth to linear growth. Cells in a culture of a phototroph transition from the exponential to the linear phase when faced with an energy limitation rather than a nutrient limitation (Sutherland et al. 1979). With a standard batch culture, this occurs as soon as the light supply to the culture becomes limiting due to light shading by the increasing number of cells. The typical rate at which photons can be absorbed and processed by the cells (in the sub-ms timescale) is much faster than the half-time of mixing of the culture. Hence, while growing, the cells in a culture are continuously exposed to transitions from light saturation to increasing light limitation. This causes the growth rate to decrease, which results in linear growth as soon as cell density increases to values at which all the incident light is absorbed. Finally, additional limitation(s) will kick in, which will cause the cells to enter the stationary phase.

Although some studies do refer to the phenomenon of a linear growth phase in batch cultures of phototrophic (micro)organisms (Cogne et al. 2005; Griese et al. 2011; Ogbonna and Tanaka 1996; Sushchik et al. 2003), more often the combined period of exponential and linear growth is referred to as cells in the culture being in the log phase, while a linear plot of the increasing OD against time is almost perfectly straight (Formighieri and Melis 2015).

For cells harvested in the exponential and in the linear growth phase, differences in mRNA transcript level and protein expression have been reported for genes related to photosynthesis and respiration (Foster et al. 2007; Ma and Mi 2005; Singh and Sherman 2006). Significantly, also biofuel production in transgenic cyanobacteria shows variation in product yields during batch growth, with some degree of growth phase dependency (Angermayr and Hellingwerf 2013; Oliver and Atsumi 2015; Savakis et al. 2013). None of these studies, however, report comparative data on changes in the physiological properties of cells harvested in the exponential and linear phases of growth.

In oxygenic photosynthesis, light harvested by antenna pigments is used to drive the photosynthetic electron flow needed for the reduction of NADP^+^. Coupled to this light-driven electron flow, a proton gradient is generated for ATP production. Respiration, which can drive ATP production in the dark, makes use of the same plastoquinone (PQ) pool as photosynthetic electron flow in cyanobacteria (Matthijs et al. 1984a, b). The largest metabolic sink of the high-free-energy carriers ATP and NADPH is carbon fixation, mediated by the enzymes of the Calvin cycle. This requires the light reactions and the so-called ‘dark reactions’ of photosynthesis (i.e., carbon fixation) to display mutually adjusted rates for optimal supply of ATP and NADPH. To secure sufficient CO_2_ availability, cyanobacteria possess an array of CO_2_ and bicarbonate transporters (Price et al. 2008; Sandrini et al. 2014). When in spite of their presence carbon limitation kicks in, surplus light energy will be wasted as heat and/or fluorescence, which in cyanobacteria involves a range of dedicated proteins associated with the two photosystems, like the orange carotenoid protein (OCP), the flavodiiron proteins, and IsiA (Allahverdiyeva et al. 2011; Bersanini et al. 2012; Havaux et al. 2005; Kirilovsky and Kerfeld 2012). Analysis of the dynamical changes in the expression level of the cyanobacterial components involved in energy generation and carbon fixation can provide insight into the tuning of both functionally coupled processes.

Light harvesting in cyanobacteria is mediated by membrane-bound, non-motile chlorophyll *a* (chl *a*)-containing protein complexes coupled to the PSII and PSI reaction centers, and by the loosely membrane-attached and motile phycobilisome antennae complexes (PBS). The PBS can migrate rapidly over the thylakoid membrane surface and transfer energy to either PSII or PSI, or to neither. In the latter case, excitation energy is lost as heat and/or emitted as PBS fluorescence (Kirilovsky and Kerfeld 2012). Energy transfer from the PBS to the photosystems can be monitored by flash freezing of the cells and analysis of the fluorescence emission spectra after specific PBS excitation at 77 K.

PSII-derived fluorescence can be recorded with the non-invasive PAM fluorimetry technique. Photon energy within PSII can be used to drive electron flow through the components of the Z-scheme, but only when the electron acceptor in the Q_A_ site (i.e., plastoquinone) is oxidized. If PQ_A_ is reduced, the PSII center is ‘closed’ and the photon energy provided to PSII by the antenna pigments will be released as heat or fluorescence. By determining the minimal (when all Q_A_ is oxidized and PSII centers are ‘open’) and maximal level of this PSII-derived fluorescence, a dynamic ‘window’ of fluorescence can be set. Within this window, the PSII-derived fluorescence can be used as an indicator for the physiological state of the cells. A low level of PSII fluorescence indicates a high availability of Q_A_ and therefore a high potential for linear electron flow. The minimal level of PSII fluorescence (F_0_) is generally derived from dark-adapted cells, while maximal fluorescence (F_M_) can be measured after subjecting cells to a strong light pulse that excites all PSII centers, which results in a transient reduction of all Q_A_ sites (Genty et al. 1989; Schreiber et al. 1986). In plants and green algae, this approach works fairly well, but in cyanobacteria things are more complicated: the cyanobacterial light-harvesting phycobilisome antenna exhibits fluorescence in the same spectral region as PSII. Furthermore, in the dark antenna binding to PSII decreases and dark respiratory electron flow influences the redox state of Q_A_ in the dark as the latter redox-active species equilibrates with the PQ pool (Campbell et al. 1998; Papageorgiou et al. 2007). PAM fluorimetry one only detects the redox state of Q_A_ (Krause and Weis 1991; Schreiber et al. 1986), but this technique is often assumed to also reflect the redox state of the PQ pool (Gotoh et al. 2010; Yang et al. 2001). However, the multitude of processes that influence both the PAM signal and the PQ pool itself make this extrapolation hard to uphold. The redox state of the PQ pool can also be determined via rapid extraction of the PQ pool itself (Schuurmans et al. 2014b). Regulation of the efficiency of photosynthesis involves both re-distribution of existing light-harvesting capacity over the two photosystems through state transitions (van Thor et al. 1998), presumably under the control of the redox state of the plastoquinone pool, and differential gene expression.

In this study, we reveal several growth phase-dependent changes of the photophysiology of *Synechocystis*, harvested in the exponential and the linear phase of growth. These growth phase-dependent adjustments are mostly the result of short-term physiological flexibility, dependent on adaptation mechanisms such as state transitions. Furthermore, the linear growth phase can be prevented or postponed by gradually increasing the incident light intensity, in parallel with the cell density in the growing culture.

## Results

### Typical growth curve of a batch culture

**Fig. 1:**
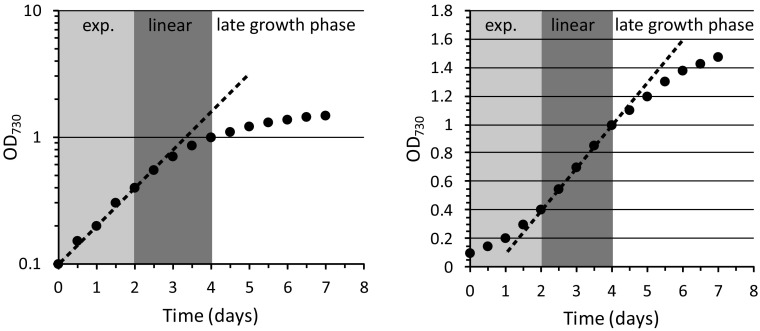
Schematic representation of a typical batch culture experiment with *Synechocystis*. Growth conditions: BG-11 medium and moderate incident light intensity. The culture displays an exponential growth phase (exp.), which gradually transits into a linear growth phase, followed by the late or stationary growth phase. On the *left*, the data are displayed on a logarithmic scale, while on the *right* they are shown on a linear scale to emphasize the presence of, and the difference between, the exponential and linear growth phases

A typical growth curve of a batch culture of a phototrophic bacterium like *Synechocystis* is schematically depicted in Fig. 1. The figure highlights the transition of exponential growth to linear growth and the slow approach to the stationary phase. Note that the transition to stationary phase can also be more abrupt and that growth phases are defined only by their shape and that the cell density at which the transitions occur varies depending on path length of the light through the culture and incident illumination intensity. The data in the left frame are also displayed on a log scale, while the right-hand frame has OD on a linear ordinate. These different ways of plotting the data show that the linear growth phase is most clearly displayed in the right-hand frame.

The effect of the gradually increasing light limitation during batch culturing on the photophysiology of *Synechocystis* cells was studied by recording several parameters in samples collected during the subsequent growth phases. From a single batch culture, we collected, at the same time points in growth, samples for 77 K fluorescence emission analysis and Western blotting, along with 100-ml aliquots for PAM fluorimetry recordings and PQ pool redox state measurements. A representative growth curve of such an experiment is displayed in Fig. S1. Table S1 also shows the optical density values at 630 and 680 nm which represent the room-temperature absorption peaks for the PBS antenna and chl *a*, respectively. The ratio of these optical density values remains essentially stable throughout the subsequent phases of growth, except for just a slight increase in the level of chl *a* (Table S1), implying that the absolute pigment content of the cells may slightly increase in the linear phase of growth.

### Gradual changes in chl *a* fluorescence during batch culturing

**Fig. 2:**
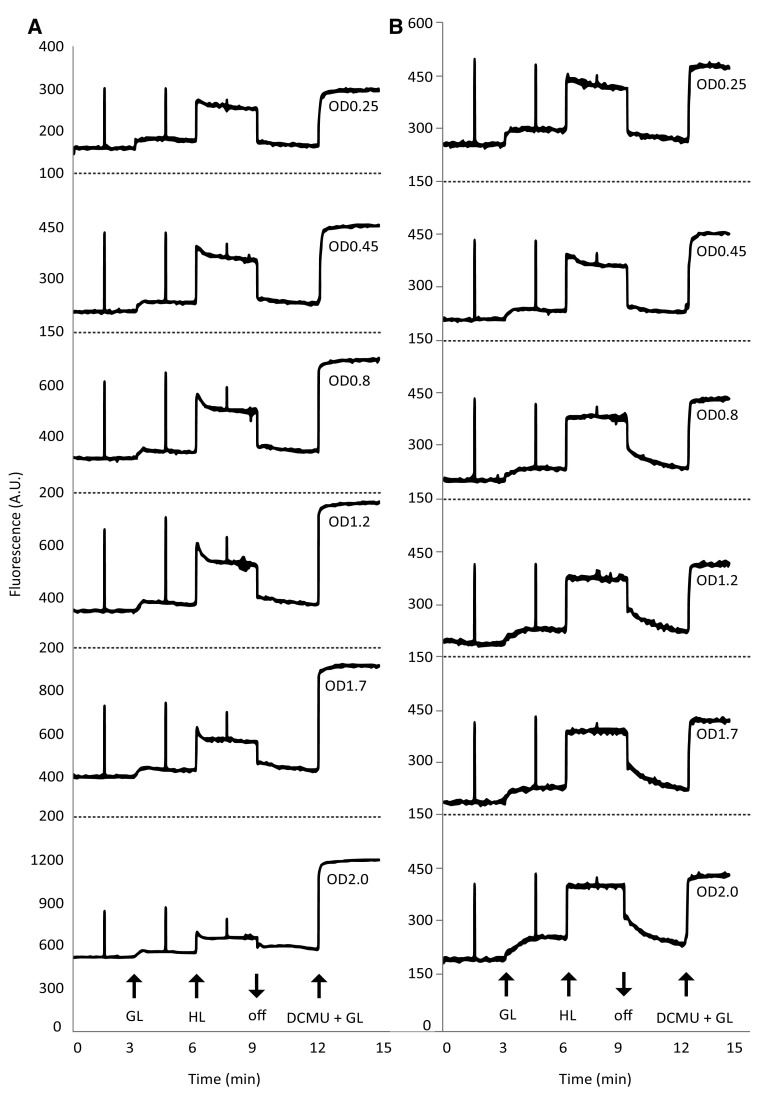
Growth phase-dependent changes in chl *a* fluorescence of *Synechocystis*. **a** PAM fluorimetry recordings of cells harvested at different growth phases. **b** PAM fluorimetry recordings of cells harvested at the same growth phase as in **a**, but with cells diluted to a standard OD730 = 0.45; at OD730 = 0.25, the recording was recalculated to 0.45. *GL* growth light intensity (30 μmol photons m^−2^ s^−1^ 655 nm LED light); *HL* high light intensity (300 μmol photons m^−2^ s^−1^ 655 nm LED light); GL+ DCMU, growth light intensity + 20 μM DCMU; *asterisk*, saturation pulse (2000 μmol photons m^−2^ s^−1^ LED light); *A.U*. arbitrary units. Data displayed are representative recordings

**Fig. 3:**
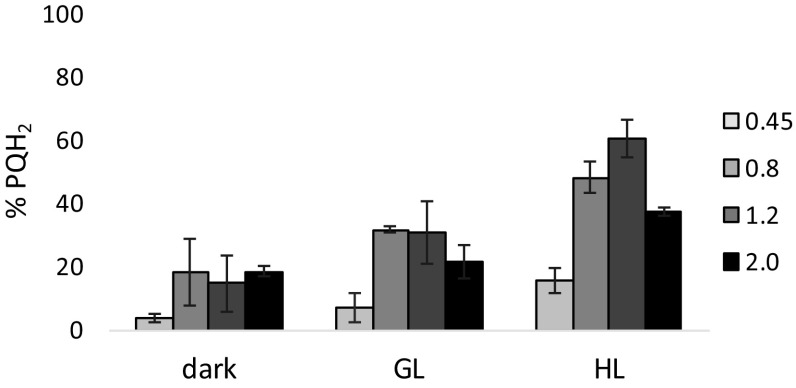
Growth phase-dependent changes in the redox state of the PQ pool of *Synechocystis*. Values are in % reduced. *GL* growth light intensity (30 μmol photons m^-2^ s^-1^ 655 nm LED light); *HL* high light intensity (300 μmol photons m^-2^ s^-1^ of 655 nm LED light). The legend specifies the OD_730_ of the culture at the time of sampling. *Error bars* indicate the range of variation of a series of duplicate measurements

For PAM fluorimetry recordings (Fig. 2; Table 1) and measurements of the redox state of the plastoquinone pool (Fig. 3), samples were dark-adapted for 30 min, followed by exposure to growth light (GL; 30 µmol photons m^−2^ s^−1^) and then to high light (HL; 300 µmol photons m^−2^ s^−1^). Cells were then returned to darkness, followed by the addition of DCMU (20 µM) and renewed exposure to growth light. Samples were either used directly from the culture (Fig. 2a) or diluted to the uniform OD_730_ of 0.45 (Fig. 2b; see “Materials and Methods” and Fig. S3 for further detail). Chl *a* fluorescence recorded from the samples that were taken directly from the culture showed a high level of fluorescence quenching at higher cell densities, yielding low fluorescence intensity after a saturation pulse, compared to the values recorded in the presence of DCMU (Fig. 2a). To acquire a more informative fluorimeter signal, pre-dilution of denser culture samples to OD_730_ = 0.45 was used. This protocol, in which cell density was equalized, permitted comparison of chl *a* fluorescence parameters in all samples regardless of the (cell) density in the culture. Interesting differences can be noticed in the derived numerical data shown in Table 1. Cyanobacteria have been reported to emit more fluorescence after a saturating pulse in the presence of low intensities of actinic light than in darkness (Campbell et al. 1998). This does not hold for samples taken from the exponential growth phase (OD 0.25 and 0.45); it only becomes apparent in the linear (OD = 0.8, 1.2, and 1.7) and in the late (OD = 2.0) growth phase (Fig. 2a, b). Also, the calculated apparent photosynthetic quantum yield of PSII (φPSII, Table 1) appears to increase somewhat with progressing growth phases and correlates with the decreasing F_0_. The PAM recordings for exponentially growing cells show a weaker response to the light treatments than cells from the linear and late growth phases (Fig. 2b). Measured in the presence of an actinic light intensity comparable to incident growth light intensity, the fluorescence increase is stronger, no relaxation response is observed at high light intensities, and the dark re-oxidation rate is much lower.

**Table 1:** Growth phase-dependent changes in chl *a* fluorescence parameters of *Synechocystis*

OD_730_	F_0_		F_M_ (dark)		F_M_ (GL)		F_M_ (DCMU)		φ PSII (max)	
	As is	At 0.45	As is	At 0.45	As is	At 0.45	As is	At 0.45	As is	At 0.45
0.25	159	262	**304**	**501**	297	489	299	492	0.48	0.48
0.45	213	213	438	438	438	438	**450**	**450**	0.53	0.53
0.8	312	204	601	**440**	628	426	**685**	437	0.55	0.54
1.2	342	191	653	417	699	**418**	**752**	417	0.55	0.54
1.7	393	189	761	419	759	**435**	**914**	423	0.57	0.57
2.0	512	195	854	410	870	**439**	**1197**	433	0.57	0.56

Variable fluorescence parameters were calculated from duplicate PAM fluorimetry traces as shown in Figure 2. The error between traces was <5%. Values from both undiluted (as is) and diluted or corrected (at 0.45) PAM recordings are shown. OD_730_, optical density at 730 nm; *F*_*0*_ minimal fluorescence after dark adaptation; *F*_M_ fluorescence after a saturating pulse in the dark or in growth light intensity (dark, GL), or after the addition of DCMU; φPSII, yield on PSII ((F_M_−F_0_)/F_M_). Values for φPSII are calculated with F_0_ and the highest recorded values for F_M_ (marked in bold)

### The plastoquinone (PQ) pool is more reduced in the linear and late phases of growth

For measurements of the redox state of the PQ pool, samples were taken during PAM measurements (see above) after dark adaptation and during growth and high-light treatment in undiluted cultures (see Figs. S2 and S3 for further detail). At OD_730_ = 0.25, the cell density proved too low for proper redox state analysis. As shown in Fig. 3, the redox state of the PQ pool shifts abruptly from almost fully oxidized in the dark and <20% reduced in the light in the exponential phase, to about 20% reduced in the dark and up to 60% reduced in the light in the linear growth phase. Surprisingly, in the stationary growth phase the redox state appears slightly more oxidized than in the linear growth phase.

### Increased energy transfer of PBS to both photosystems in the linear and the late growth phase

**Fig. 4:**
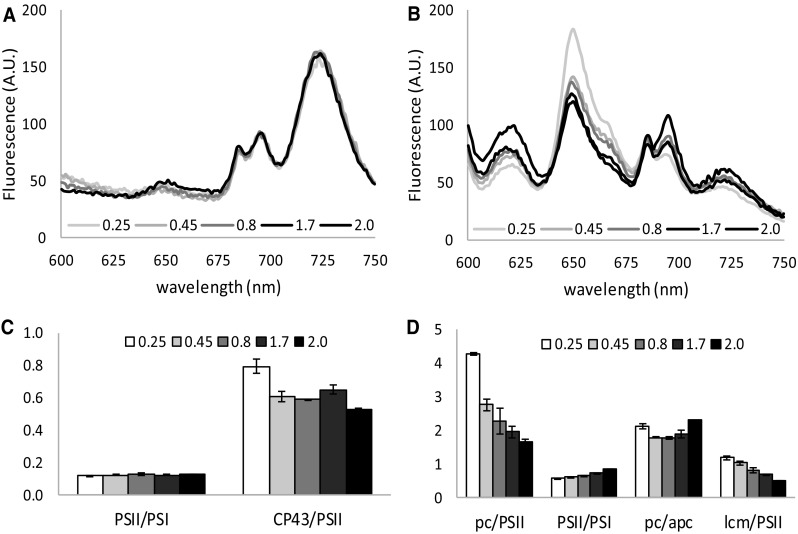
Growth phase-dependent changes in light adaptation, as demonstrated with 77K fluorescence emission spectra of *Synechocystis* with 435 nm (**a, c**) and 590 nm (**b, d**) excitation. Shown are fluorescence emission spectra at different optical densities taken in growth light (GL) conditions, normalized to the area under the curve and the ratios between several peaks. Peak area was determined using the peak fitting add-in for excel from http://www.chem.qmul.ac.uk/software/expfit.htm on raw data. Peak at 620 nm, measurement artifact (see M&M); 655 nm, pc; 665 nm, apc; 686 nm, lcm; 696 nm, PSII; 720 nm, PSI. Data were derived from biological duplicates and technical triplicates; shown is the average with standard deviation

Samples for 77 K fluorescence emission analysis were taken directly from the cell culture under growth light intensity (30 µmol photons m^− 2^ s^− 1^). Fluorescence spectra (Fig. 4a, b) were recorded at 77 K with the excitation wavelength being set at 435 nm (chl *a*) and 590 nm (PBS). The ratios between peak areas are shown in Fig. 4c, d to show the fluorescence signal dynamics in more detail. Figure 4a, with 435 nm excitation, shows that the apparent PSII/PSI ratio is stable throughout growth, with perhaps a slightly lower value in the stationary phase, as reflected in Fig. 4c. It can be observed from Fig. 4b with 590 nm excitation that cells collected from the phase of exponential growth display high levels of phycocyanin (pc, 655 nm) and allophycocyanin (apc, 665 nm) fluorescence. Furthermore, one can see that as the culture grows more and more dense, PBS fluorescence decreases and PSII (696 nm) and PSI (720 nm) fluorescence increase. This increase in 696 and 720 nm fluorescence indicates higher levels of energy transfer from the PBS to the two photosystems in the linear and late growth phases. It can also be observed from Fig. 4d that the energy transfer of the PBS to the two photosystems increases significantly (pc/PSII) in the transition from the exponential to the linear growth phase, with a slight preference for PSII, as reflected by the slight increase in the PSII-to-PSI fluorescence ratio. Although the total PBS fluorescence decreases, the ratio between pc and apc fluorescence remains stable (pc/apc). On the high-energy side of PSII, with both chl *a* and PBS excitation, an additional peak (at 686 nm) is visible. In literature, this peak has been attributed to three different proteins: the iron-inducible IsiA (van der Weij-de Wit et al. 2007), the chl *a*-containing subunit CP43 of PSII (Groot et al. 1999), and the PBS terminal emitter Lcm (Wilson et al. 2007). Because *isiA* expression is unlikely to occur under the growth conditions used here (low-light and iron-replete) and because of the nature of the excitation wavelengths used, we attribute the peak with 435 nm excitation to CP43 and to Lcm with 590 nm excitation.

### Protein expression levels of the two photosystems and of the RuBisCO enzyme

**Fig. 5:**
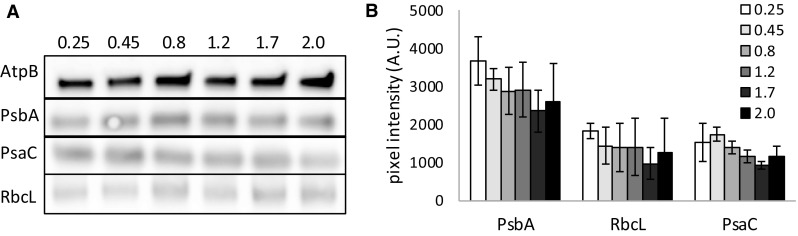
Western blots showing the expression levels of selected proteins involved in photosynthesis at different growth phases in *Synechocystis*. **a** Protein immunoblots showing the content of PsbA, PsaC, RbcL, and AtpB in *Synechocystis*. Ten micrograms of total protein were loaded in *each lane*. **b** Pixel intensity of protein immunoblots normalized to AtpB levels. Three individual blots for each protein were analyzed with ImageJ; values shown are averages with standard deviation

Because 77 K fluorescence emission ratios are only an indirect indication of the true PSII/PSI ratio, we wanted to test if the physiological changes observed in the photophysiology of *Synechocystis* could still be caused by changes in the level of protein expression of the components of the photosynthetic machinery and/or RuBisCO, for this quantitative Western blotting was used. Cell samples were taken directly from the cell culture and lysed using a French press. Cell-free extracts were analyzed by Western blotting with specific antibodies elicited against PsbA (D1 subunit of PSII), PsaC (subunit of PSI), and RbcL (large subunit of RuBisCO). As an internal standard, the levels of AtpB were determined. The levels of the tested proteins barely change in these samples (Fig. 5a). In Fig. 5b, the average stain density of three separate blots is shown, and from these data a slight downward trend may be observed along with the transition from exponential to linear growth for all three tested proteins.

### Onset and cause of the phase of linear growth

**Fig. 6:**
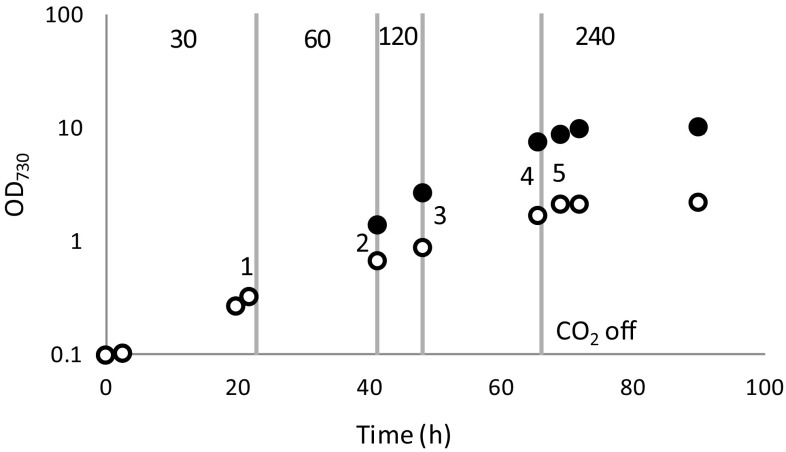
Growth curve of *Synechocystis* in BG-11 medium in two illumination regimes with 10 mM TES-KOH pH 8 and air + 1% CO_2_ bubbling. *White dots* cultures growing at 30 μmol photons m^−2^ s^−1^. *Black dots* cultures growing under increasing incident light intensity conditions as indicated by the *vertical gray lines* and the light intensity marked at the *top* in μmol photons m^−2^ s^−1^. *Gray dotted line*, CO_2_ supply stopped for all cultures, to induce carbon limitation and a transition to the stationary phase. Shown is the average of four individual batch cultures; standard deviations were too small to be visible beyond the size of the data points. The *numbers* in the figure refer to the sampling points at which cells were analyzed with qPCR (see Fig. 7)

To provide evidence that the linear phase of growth in a regular batch culture is caused by light limitation, a batch culture was exposed to light that increased in intensity in parallel to the increasing cell density, to extend the length of the exponential phase (Fig. 6, black dots). Diminishing the decrease in light intensity perceived per cells in this way significantly extends the length of the exponential growth period, to the extent that under the selected conditions the exponential growth phase fully replaces the linear growth phase. By changing the gas phase of the cultures from CO_2_-enriched air to nitrogen only (via bubbling with nitrogen gas), cultures can rapidly be brought to carbon limitation, independent of the light supply, resulting in a transition to the stationary phase. This experiment shows that the observed transition from exponential to linear growth indeed is caused solely by light limitation, while a transition to the stationary phase is caused by nutrient limitation.

### Lower levels of mRNA for carbon uptake systems in the linear growth phase

**Fig. 7:**
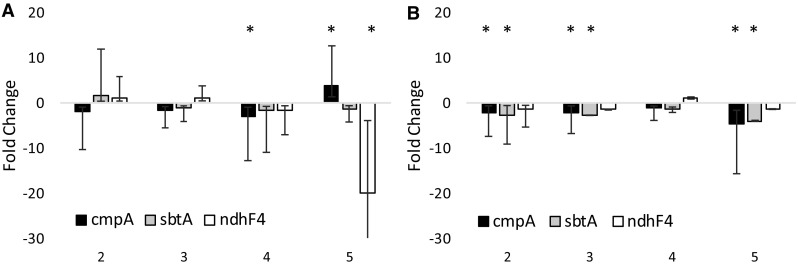
Fold change in the expression levels of three Ci transporters at different stages of growth in *Synechocystis*, analyzed with qPCR. **a** Changes in expression in cultures growing exponentially at increasing light intensities (*black dots* in Fig. 6) relative to the expression levels at the lowest light intensity (#1 in Fig. 6). **b** Changes in expression in linearly growing cultures at low light intensity (*open dots* in Fig. 6) relative to the expression levels when growth was still exponential (#1 in Fig. 6). Significant change is marked with an *asterisk. cmpA* stands for the subunit of the ATP-dependent HCO3 transporter; *sbtA*, Na^+^-dependent HCO3 transporter; *ndhF4*, subunit of NADPH-dependent CO2 transporter. Expression levels were normalized to the expression of the housekeeping gene *rnpB*. Shown are the averages of biological duplicates with the relevant error range

Here the level of transcription of several carbon uptake systems was investigated, since faster growth will be supported by higher rates of carbon fixation. We chose to test the low C_i_-inducible ATP-dependent bicarbonate and carbonic acid transporter *cmpA*, the low C_i_-inducible Na^+^-dependent bicarbonate transporter *sbtA*, and an NADPH dehydrogenase involved in constitutive CO_2_ uptake *ndhF4*. For this experiment, samples were taken for qPCR from the experiment depicted in Fig. 6. The order of sampling is depicted in this figure: (#1) before the light intensity was increased, i.e., when all parallel cultures were still subject to the same regime; these samples were used as the reference. Next, the samples were taken in each illumination condition, just before the conditions were changed (#2, 3, and 4), followed by one last sample in the (intentionally induced) carbon-limited stationary phase (#5). The qPCR results show that the cultures that were enabled to continue exponential growth by raising the incident light intensity show little to no (downward) change in the expression levels of the different carbon uptake systems; taking away the CO_2_ supply gives rise to a sharp response: less transcript for the constitutive transporter NdhF4 and more of the inducible transporter CmpA (Fig. 7a). In contrast, in the control culture where the light intensity was kept constant and the culture transitioned to linear growth, a consistent decrease in the expression level of the tested carbon uptake systems was revealed (Fig. 7b).

## Discussion

Heterotrophic microorganisms that are grown in batch culture have three distinct growth phases: an optional lag phase, a log phase (also known as the exponential growth phase) in which there is no nutrient limitation, and a nutrient-limited stationary phase in which cells do not, or barely, grow. Photoautotrophic microorganisms on the other hand display an additional phase known as the linear growth phase, marked by energy limitation (Sutherland et al. 1979), often in the form of the limited and inhomogeneous availability of the substrate light. Proof that decreased light availability is the effector for the transition of the exponential to the linear growth phase is provided here in an experiment in which, along with cell density, the light intensity was raised, which prolonged the duration of the exponential growth phase and even prevented the transition because those cells directly entered the stationary phase.

Growth phase-dependent changes of gene expression for the cyanobacterium *Synechocystis* sp. PCC 6803 were earlier reported by Foster et al. (2007), who observed in a microarray study that the transcript level of many genes related to photosynthesis decreases in the linear growth phase. A year earlier, it was found that the expression levels of the *isiA* and *psbA* genes fluctuate during growth with high expression levels in the exponential phase (Singh and Sherman 2006). The present study adds to this that transitions between subsequent phases of growth are accompanied by physiological changes affecting photosynthesis. Chl *a* fluorescence, recorded by PAM fluorimetry, showed increased levels of emitted fluorescence in response to light, as growth progressed, which is accompanied by a decrease in the dark re-oxidation rate of PSII. This increase in fluorescence could reflect a more reduced state of Q_A_, and perhaps also of the PQ pool, an increase in energy transfer to PSII, an increase in PSII abundance, or any combination of these processes. To distinguish between these options and to determine whether the recorded changes resulted mostly from physiological adaptation or from changes at the level of transcription or translation, we used various analytical techniques: Firstly, 77 K fluorescence emission spectroscopy was performed, which provided insight into the energy transfer of the phycobilisome light-harvesting antennae to the two photosystems. The data obtained show that energy transfer from these antennae to PSII increased when cell density increased, which is associated well with the observed shift of the plastoquinone pool to a more reduced state in the linear growth phase. The stable PSII-to-PSI ratio inferred from 77 K fluorescence emission spectroscopy (Fig. 4a) implies that this increase in PSII-related fluorescence with PBS excitation light is caused by post-transcriptional changes and not by an increase in the expression level of the PSII protein.

Additional insight into possible changes in the expression level of photosynthesis-related proteins was obtained via quantification of some key proteins by quantitative Western blotting analysis, using PsbA for PSII, PsaC for PSI, and RbcL for RuBisCO to survey possible changes in carbon fixation. Although Western blotting revealed that a slight downward trend was observed for the expression level of all proteins tested as growth progresses, their relative decrease was comparable, indicating that there was no change in PSII-to-PSI reaction center ratio, as already implied with 77 K fluorescence analysis, which could have provided an alternative explanation of the observed reduction of the redox components of the PSII acceptor side and of the PQ pool. Even though the changes reported here are only minor, they fit well with earlier reports on changes in gene transcription level between exponentially and linearly growing cells, which showed a mild decrease in the expression of *rbcL* (1.5-fold) and fairly stable expression of *psaC* and *psbA* (<1.3-fold change) (Foster et al. 2007), although Singh and Sherman (2006) earlier reported higher levels of *psbA* transcripts in the exponential phase.

Analysis of the transcript abundance of several inorganic carbon transporters, i.e., the low C_i_-inducible *cmpA* (Omata et al. 1999) and *sbtA* (Shibata et al. 2002) and the constitutive, C_i_ concentration-insensitive *ndhF4* (McGinn et al. 2003), revealed that the cells that exhibit linear growth show a decrease in transcript abundance for the inducible *cmpA* and *sbtA* transcripts. This corresponds well with a higher carbon demand in exponentially growing cells compared to cells that display linear growth. From the exponentially growing cultures, only the transcript of *cmpA* shows a significant decrease in abundance at time point 4 (Fig. 7a); this likely represents the start of the linear phase as is also visible in Fig. 6. Under the carbon-limiting conditions induced here, the expression level of most tested carbon uptake systems is lower than that in growing cells. This demonstrates that stationary phase induction has a net negative effect on gene expression, which also apparently includes carbon uptake systems under carbon-limited conditions.

An interesting trend in the chl *a* fluorescence parameters is the increasing apparent photosynthetic yield (φPSII, Table 1) as growth progresses. Cultures in the exponential phase have a somewhat higher F_0_ value compared to cells from the linear and late growth phases in diluted cultures. 77 K fluorescence emission spectra show high levels of PBS fluorescence in the exponential phase, which strongly and continuously decreases in the linear and late growth phases. Additionally, Table S1 shows a slight decrease in the PBS-to-cell density ratios in the stationary phase implying a slight decrease in the total PBS content. PAM fluorimetry on cyanobacteria is compromised by several aspects intrinsic to the photosynthetic machinery of cyanobacteria, making PAM data more difficult to interpret in these organisms (Schuurmans et al. 2015). The most influential one of these aspects is PBS background fluorescence which causes underestimation of φPSII by artificially increasing perceived F_0_ values (Campbell et al. 1998; Schuurmans et al. 2015). The change in φPSII observed here is most likely another example of PBS background fluorescence interference rather than an actual increase in φPSII with decreasing growth rate. A second point of interest is that cyanobacteria are described to be in state II in the dark, with little binding of the PBS to PSII (Campbell et al. 1998). In ambient (growth) light, the cells then go to state I, with strong binding of the PBS to PSII. Because of this, saturation pulses given in growth light result in higher levels of chl *a* fluorescence, due to better energy transfer to PSII, than saturation pulses given in the dark (Campbell et al. 1998; Papageorgiou et al. 2007). Yet, in this study we only observe this behavior in the linear and late growth phases (OD_730_ ≥ 1.2). This behavior (no increase in F_M_′ in the light) has been reported for *Synechocystis* before (Goosney and Miller, 1997) and from this the conclusion was then drawn that *Synechocystis* does not enter state II in the dark (Campbell et al. 1998). However, from the 77 K fluorescence emission spectra shown in this study it appears that the proposed state I transition in the light only occurs in the linear and late growth phases, where PBS binding to PSII is strong, while cells in the exponential phase remain in state II, with low levels of PBS binding to PSII and therefore no increase in energy transfer to this photosystem. Although this opposes the conclusion drawn earlier, it does still concur with the idea that no state transition takes place in exponentially growing cultures of *Synechocystis*, which means no increase in $\text{F}_{\text{M}}^{^{\prime }}$ is observed in the light.

PAM fluorimetry is a highly sensitive technique, of which the results are very strongly influenced by illumination and growth conditions, both prior to and during measurements, as well as to genetic variations in, for instance, pigment composition and excitation-dissipating mechanisms. So for proper interpretation of PAM results (pre)growth conditions should always be clearly stated and caution is advised on extrapolation of PAM data of one strain to other (lab) strains of the same species, which are known to display strain-to-strain differences, and especially on extrapolation to other species. Recently, more species-specific variation in the state transitions of cyanobacteria has been described (Misumi et al. 2016). These authors compared six cyanobacterial strains, originating from a terrestrial, an aquatic, or a symbiotic habitat is described. The terrestrial species show the most prevalent state II in the dark, while the symbiotic species remain in state I. As an aquatic organism, *Synechocystis* shows intermediate behavior. Here the original habitat of an organism may very well be the cause of the characteristics of its state transitions: with a much more cautious (state II) energy transfer to PSII in species that are adjusted to high, stressful, incident light intensities (i.e., the terrestrial habitat), compared to species receiving lower, and more stable, light intensities (i.e., symbionts). Batch cultures of an aquatic cyanobacterium like *Synechocystis* essentially grow from a high-light environment (light-saturated exponential growth) toward a low-light environment (light-limited linear growth) with a matching shift in state transition from state II in high light to state I in low light.

The changes in the photophysiology that are observed in this study appear to be mainly based on the adaptation of the antennae to photosystem connectivity. In the exponential phase, plentiful light gives rise to photon energy dissipation as antenna fluorescence, without signs of lesser transcription or expression of phycobilisome proteins. This confirms the expression studies conducted by Singh and Sherman (2006) and Foster et al. (2007). The changes observed in this study in the chl *a* fluorescence response can be explained by the changes in energy transfer in the antenna and the redox state of the plastoquinone pool, but do not explain why these changes occur. State transitions in literature are often described to be regulated by the redox state of the PQ pool (Allen et al. 1981; Mullineaux and Allen 1990), although several studies report on the involvement of the cytochrome b_6_f complex (de Vitry et al. 2004; El Bissati and Kirilovsky 2001; Vener et al. 1997; Vernotte et al. 1990). When energy-consuming processes such as carbon fixation, biomass formation, and nutrient acquisition are limiting growth, then the rate of input of electrons into the PQ pool should exceed the rate of output, leading to a net reduction of the PQ pool. And vice versa, when energy production (light and respiration) is limiting, output should exceed input leading to a net oxidation. Yet this does not happen; under light-saturated exponential growth conditions, energy transfer to PSII, and therefore PSII activity, is lowered to prevent strong reduction of the plastoquinone pool, leading to a net oxidation of the PQ pool. This is consistent with our earlier findings that, under carbon-limiting conditions, the plastoquinone pool is more oxidized than under carbon-replete conditions (Schuurmans et al. 2014b). This also implies that the light state of the cell and concomitant PSII activity determine the redox state of the PQ pool and not the other way around. To properly regulate and maintain the balance between energy consumption and production, the cell must monitor both these processes. If the redox state of the PQ pool is indeed largely defined by PSII activity, then this pool is not a suitable detector of the energy balance. The cytochrome b_6_f complex, however, is influenced by both PQH_2_ (input) and plastocyanin (PC) and cytochrome *c* availability (output). Studies on chloroplasts of plants and algae suggest that binding/oxidation of PQH_2_ in the Q_0_ site of the cytochrome b_6_f complex triggers a conformational change which in turn triggers thylakoid phosphorylation via the dedicated kinases STN7/Skk7 and then a state II transition (de Vitry et al. 2004; Rochaix 2007; Vener et al. 1997). In cyanobacteria, however, such kinases (and for that matter: also phosphatases) have not been identified. Considering the sidedness of the cyanobacterial thylakoids, we think that it is therefore more likely that the state transitions in cyanobacteria are triggered by alteration of the net charge of the species bound to the Q_i_ site of the cytochrome b_6_/f complex through electrostatic effects (of, e.g., PQ^−^ and PQH^−^). Because quinol turnover is much faster than antenna migration (de Vitry et al. 2004), a stable state II of the light-harvesting antenna requires high levels of charge accumulation of the Q_i_ site and a stable state I requires it to be neutral. If this is correct, then the data described in this study together with our earlier findings (Schuurmans et al. 2014b) suggest that even relatively low levels of PQH_2_ (10% of the total pool, Fig. 3) are sufficient to achieve this, and regulation depends on the turnover rate of the Q_i_ site, fueled by PC and cytochrome *c* availability and PSI activity. In the dark, the minimal required level of PQH_2_ for charge accumulation may be even lower because PSI is not active under these conditions, allowing for the cyanobacterial signature state II transition to occur in the dark with only the respiratory supply of plastoquinol (≥5% PQH_2_, Fig. 3).

In recent years, the interest in phototrophic microorganisms for biotechnological applications has increased strongly (dos Santos et al. 2014; Hellingwerf and Teixeira De Mattos 2009; Nogales et al. 2012). Their independence from arable land and the higher photosynthetic yield on light compared to plants make algae and cyanobacteria ideal candidates for CO_2_-based biomass and biofuel production (Schuurmans et al. 2014a). Improving growth and biomass or product formation in large-scale cultures is a main priority in this field of research. Several studies with transgenic biofuel-producing cyanobacteria have already shown that product yields and carbon partitioning to product can differ between growth phases (Angermayr and Hellingwerf 2013; Oliver and Atsumi 2015; Savakis et al. 2013). The variations in product yield over the course of growth in batch can also differ between different products of interest (compare Angermayr and Hellingwerf 2013 with Savakis et al. 2013) and determining the optimal growth phase for harvesting of any particular product could be very helpful in optimizing production and product yield.

## Materials and methods

### Strains and culture conditions

*Synechocystis* sp. PCC 6803 was grown as a batch culture in a photobioreactor (Huisman et al. 2002) at a temperature of 30 °C. Growth was in continuous white fluorescent light (30 µmol photons m^− 2^ s^− 1^ incident light) in BG-11 mineral medium (Rippka et al. 1979) with 10 mM Na_2_CO_3_. Mixing was established with a stream of sparged air, enriched with 1% CO_2_ at a rate of 20 L h^− 1^. In the experiment where the exponential phase was extended, *Synechocystis* sp. PCC 6803 was grown as a batch culture in a multicultivator (PSI) in BG-11 mineral medium with 10 mM TES-KOH pH 8; the culture was bubbled with 1% CO_2_-enriched air. Growth was in continuous white fluorescent light which was increased manually in parallel to increasing cell density.

### PAM fluorimetry

100-ml aliquots of the *Synechocystis* cultures were taken at different times during growth, thus covering different growth phases. At higher cell densities, chl *a* fluorescence was recorded from samples as is and from samples that were diluted to an OD_730_ = 0.45 prior to recording. At cell densities below OD_730_ = 0.45, chl *a* fluorescence was recorded as is and recalculated to OD_730_ = 0.45 for the comparison shown in Fig. 2b. The collected samples were placed in a small flat-panel vessel with a light path of 3 cm. The vessel was placed in between two 660 nm LED light sources to ensure a constant light climate inside the vessel. The culture was mixed by a stream of sparged air, enriched with 1% CO_2_ at a rate of 10 L h^− 1^ and by a magnetic stirring device. The monitoring optical fiber of the PAM-100/103 fluorescence monitoring instrument (Walz, Germany) was placed against the side of the vessel, perpendicular to the light sources. The vessel was equipped with a rapid sampler, used for PQ redox state determination, as well as a syringe with a long needle for rapid DCMU addition (see Fig. S2 for a schematic of this set-up). At the start of the experiments, the cultures were dark-adapted for 30 min, after which the cultures were exposed to 3 min of illumination at growth light intensity (30 µmol photons m^−2^ s^−1^), followed by 3 min of illumination at high light intensity (300 µmol photons m^−2^ s^−1^). At 1.5 min before the light conditions changed, a saturation pulse (2000 µmol photons m^−2^ s^−1^), generated by the same LED lamps that secured the actinic light, was applied to determine F_M_. After the light treatments, the culture was incubated in the dark for 3 min followed by a final treatment with 20 µM 3-(3,4-dichlorophenyl)-1,1-dimethylurea (DCMU, Sigma) at growth light intensity (see Fig. S3 for a schematic representation of this regime).

### PQ pool redox state measurements

Samples for the determination of the PQ pool redox state were taken during the PAM fluorimetry experiment, as described above and indicated in Fig. S3. Samples were taken shortly before application of the saturating pulse in the dark-adapted state and during growth-light and high-light illumination. During dark adaptation, before chl *a* fluorescence measurements, the samples were taken and fully reduced with NaBH_4_ to determine the size of the PQ pool. For plastoquinol (PQH_2_) extraction, detection, and subsequent PQ pool redox state analysis, the method described earlier in Schuurmans et al. (2014b) was used. In short, the samples were quenched in an ice-cold mixture of 1:1 (v/v) methanol/petroleum ether (PE), followed by vortexing and centrifugation. The top PE phase was transferred to an N_2_-flushed glass tube and the PE extraction was repeated. PE phases were pooled and dried under a continuous stream of N_2_ gas. Dried samples were resuspended in hexanol and the PQH_2_ was detected using HPLC with fluorescence detection (excitation/emission was set at 290/330 nm). The PQ pool redox state was calculated using the PQH_2_ values obtained from fully (NaBH_4_) reduced samples as a 100% reference.

### 77 K fluorescence emission analysis

For 77 K fluorescence analysis, the samples were taken at different growth phases and diluted to a final OD_730_ of 0.15 in BG-11 medium with glycerol (final concentration 30% (v/v)) and immediately frozen in liquid nitrogen. The samples were analyzed in an OLIS 500 spectrofluorimeter, equipped with a Dewar cell. This fluorimeter always generates a peak within the first 50 nm of the recorded emission spectra, irrespective of the excitation wavelength or the range of emission wavelengths recorded. Because this phenomenon is consistent and independent of the sample conditions, it has been deemed an artifact; in this study, the artifact is visible at 520 nm in the spectra recorded with 435 nm excitation (data not shown) and at 620 nm in the spectra recorded with 590 nm excitation. Chl *a*-specific excitation light was used at 435 nm and fluorescence emission spectra were recorded between 500 and 750 nm. PBS-specific excitation light was used at 590 nm, and fluorescence emission spectra were recorded between 600 and 750 nm. In this wavelength domain, the phycobilisomes (655 nm, pc and 665 nm, apc), PSII (696 nm), and PSI (720 nm) show well-separated emission peaks. Peak deconvolution was performed using the peak fitting add-in for excel from the site: http://www.chem.qmul.ac.uk/software/eXPFit.htm on the different peaks, in order to assay the degree of coupling of the PBS to PSII and PSI.

### Western blotting

Cell samples were taken from the *Synechocystis* culture at different growth phases to a final OD_730_ of 60 in 1 ml. Samples were spun down (10 min, 3500 g) and resuspended in 50 mM MES buffer, pH 6.5. The cells were broken by four passages through a French pressure cell at 12,000 p.s.i. The protein concentration of the cell-free extracts was determined with a Pierce BCA assay and 10 µg of protein was loaded onto 15% SDS-PAGE gels. Gels were run for 2 h at 10 mA per gel and proteins were transferred to nitrocellulose membranes by wet blotting at 50 mA overnight. Blots were stained with antibodies elicited against AtpB and PsbA or PsaC. Blots with antibodies against RbcL were conducted without AtpB because these proteins are of approximately the same size. For each antibody at least 3 blots were made. GARPO (goat anti-rabbit peroxidase) was used as the secondary antibody. Visualization was achieved using a supersensitive chemo-luminescence kit (Thermo) and a LiCor Odyssey FC Imager. Band intensity was determined in triplicate using ImageJ software. Band intensities of the different proteins were normalized to the band intensity of AtpB. RbcL band intensity was normalized to AtpB from AtpB-PsbA blots which were conducted simultaneously.

### Transcript analysis by qRT-PCR

*Synechocystis* cells were harvested at the different time points indicated in Fig. 6, and rapidly concentrated to a uniform final OD_730_ = 3.3 in 1 ml and opened by bead-beating with intervals for cooling, cell debris was removed by centrifugation. Prior to use, the glass beads were washed in 4 N HCl and ethanol and autoclaved twice. RNA was isolated using the RNeasy mini kit (Qiagen) and further used to prepare cDNA with the RevertAid First-Strand cDNA 13 Synthesis Kit (Thermo Scientific). RNA quantity was determined on a NanoDrop 1000 spectrophotometer (Thermo Scientific) and quality was assessed on a 1% agarose gel to confirm RNA integrity. For cDNA formation, the RevertAid First-Strand cDNA Synthesis Kit (Thermo Scientific) was used with the M-MuLV Reverse Transcriptase and random hexamer primers. The cDNA yield was analyzed by qRT-PCR in an Applied Biosystems 7300 Real-Time PCR system using the Power SYBR^®^ Green PCR Master Mix (Life Technologies) employing gene-specific primers. Gene-specific primers were designed with Primer3 software (Life Technologies) (Table S2) for the ATP-dependent HCO_3_ transporter subunit *cmpA*, the Na^+^-dependent HCO_3_ transporter *sbtA*, the NADPH-dependent CO_2_ transporter subunit *ndhF4*, and the housekeeping gene *rnpB* (Pinto et al. 2012). The relative abundance was calculated and normalized to *rnpB* which is used as a housekeeping control (Pinto et al. 2012) in samples from different conditions or different mutant strains (ΔΔCt method). Significance was tested on normalized Ct values with a Student’s T test.

## Electronic supplementary material

Below is the link to the electronic supplementary material.

Supplementary material 1 (DOCX 128 KB)

## Abbreviations

PSI: Photosystem 1
PSII: Photosystem 2
PBS: Phycobilisome
PC: Phycocyanin
PQ: Plastoquinone
DCMU: 3-(3,4-Dichlorophenyl)-1,1-dimethylurea
OD: Optical density
