# Supplementary material for: Transition from exponential to linear photoautotrophic growth changes the physiology of Synechocystis sp. PCC 6803
Source: Photosynth Res. 2017 Jan 20;132(1):69–82. doi: 10.1007/s11120-016-0329-8 (PMC5357262; doi:10.1007/s11120-016-0329-8)
Supplement: Supplementary file 1 — Supplementary material 1 (DOCX 128 KB) [file 11120_2016_329_MOESM1_ESM.docx]

# Supplementary materials:


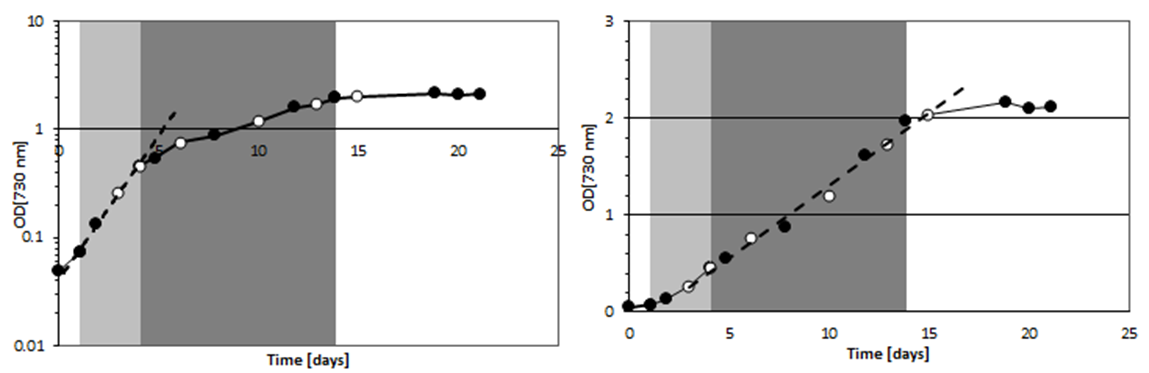


Figure S1: Batch culture sampling scheme. Growth curve of *Synechocystis* WT growing in batch with incident white light at 30 µmol photons m^-2^ s^-1^ in BG-11 medium with 10 mM Na_2_CO_3_, bubbled with air + 1% CO_2_ at 30 °C. The open circles indicate the time points at which samples were taken for further measurements, at the closed circles only the optical density was measured.

| Time (days) | OD_630_ | OD_680_ | OD_730_ | 630:680 | 630:730 | 680:730 |
| --- | --- | --- | --- | --- | --- | --- |
| 2.9 | 0.36 ± 0.003 | 0.34 ± 0.003 | 0.26 ± 0.003 | 1.07 ± 0.013 | 1.40 ± 0.022 | 1.32 ± 0.020 |
| 4.0 | 0.65 ± 0.004 | 0.61 ± 0.003 | 0.46 ± 0.002 | 1.06 ± 0.008 | 1.40 ± 0.011 | 1.32 ± 0.010 |
| 6.1 | 1.09 ± 0.004 | 1.04 ± 0.002 | 0.78 ± 0.005 | 1.05 ± 0.004 | 1.40 ± 0.010 | 1.34 ± 0.009 |
| 10.0 | 1.60 ± 0.007 | 1.56 ± 0.008 | 1.22 ± 0.008 | 1.04 ± 0.009 | 1.39 ± 0.015 | 1.34 ± 0.015 |
| 12.9 | 2.34 ± 0.024 | 2.29 ± 0.02 | 1.69 ± 0.017 | 1.02 ± 0.014 | 1.38 ± 0.020 | 1.35 ± 0.018 |
| 14.9 | 2.72 ± 0.037 | 2.66 ± 0.037 | 2.04 ± 0.037 | 1.03 ± 0.020 | 1.34 ± 0.030 | 1.30 ± 0.030 |

Table S1. Optical density values at 630 nm (PBS), 680 nm (chl a) and 730 nm (density) of *Synechocystis* WT growing in batch with incident white light at 30 µmol photons m^-2^ s^-1^ in BG-11 medium with 10 mM Na_2_CO_3_, bubbled with air + 1% CO_2_ at 30 °C. The data matches the growth curve shown in figure S1 and only the points at which further sampling occurred are shown.

1

2

3

4

7

5

6

A

8

8

4

23cm

23cm

3cm

B

2

6

Figure S2: schematic representation of the PAM-set-up. A: front view; B: side view. 1, inlet for 1 % CO_2_ enriched Air supply (10 L.h^-1^); 2, Syringe; 3, Rapid sampler; 4, Flat panel vessel; 5, Hollow glass rods; 6, location of the PAM light guide fiber; 7, Stirring magnet; 8, LED panels.


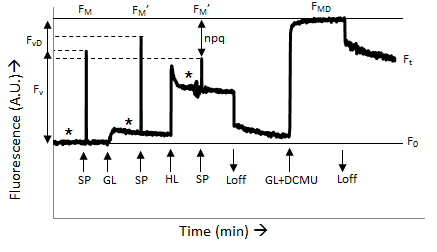


Figure S3: Schematic representation of results of measurement of variable chl *a* fluorescence. Typical example of a chl *a* fluorescence trace of *Synechocystis* as acquired in this study. SP, saturating pulse; GL, growth intensity actinic red light (30 µmol photons m^-2^ s^-1^); HL, high actinic red light (300 µmol photons m^-2^ s^-1^); Loff, actinic light is switched off; GL+DCMU, 30 µmol photons m^-2^ s^-1^ red light + 20 µM DCMU. F_t_, fluorescence level at any given time; F_0_, fluorescence level in the dark; F_M_, level of fluorescence acquired with a saturating pulse in the dark; F_M_’, level of fluorescence acquired with a saturating pulse in the light; F_MD_, level of fluorescence acquired in the presence of DCMU and GL. F_V_, variable fluorescence (F_M_-F_0_); F_VD_, variable fluorescence (F_MD_-F_0_); npq, non-photochemical quenching. Stars, sampling points for determination of the PQ redox state.

| Name | Sequence 5' - 3', forward primer | Sequence 5' - 3', reverse primer |
| --- | --- | --- |
| cmpA | ATCCTGACACCGATATTGACCTACT | CGCATGCCCTGGACTGTT |
| sbtA | CCGGAAGATAATCGGGTCAA | GGCAGGGCCTTGTAAACTTTCT |
| ndhF4 | CCCCCATTTCAGTGATATTTTGA | TGTTATTTATGAGCATTGGTTCGATT |
| rnpB | GCGCACCAGCAGTATCGA | CCTCCGACCTTGCTTCCAA |

Table S2. Primers used for qPCR analysis
